# An Atypical Case of a Common Pregnancy Issue: Appendicitis-Like Hyperemesis Gravidarum

**DOI:** 10.1155/2020/6959605

**Published:** 2020-07-19

**Authors:** Janice Chung, Ryan P. Berryman

**Affiliations:** ^1^Creighton University, School of Medicine, Phoenix Regional Campus, Phoenix, AZ, USA; ^2^Department of Internal Medicine, Saint Joseph's Hospital and Medical Center, Phoenix, AZ, USA

## Abstract

Hyperemesis gravidarum (HG) is a severe subtype of nausea and vomiting in pregnancy (NVP) that typically affects women in their first trimester of pregnancy. Because HG is a diagnosis of exclusion, a thorough work-up ruling out organic causes must be performed. Herein, we describe a case of a pregnant woman with HG who presented with intractable pain mimicking appendicitis. While her clinical picture and ultrasound findings were only mildly consistent with appendicitis, the only therapy that provided pain relief was empiric antibiotic treatment that was prescribed due to a mildly elevated procalcitonin of 0.61. Thereby, the detection and treatment of concurrent organic causes is important as it may provide significant symptomatic relief in cases of concurrent HG.

## 1. Introduction

Hyperemesis gravidarum (HG) is a severe subtype of nausea and vomiting in pregnancy (NVP) that is estimated to affect 0.3–2% of pregnancies [1, 2]. It is a common reason for admission to the hospital in the first trimester of pregnancy [2]. HG typically begins in the first trimester of pregnancy, likely due to increasing amounts of hormones, and usually ends in the second trimester, although some cases have lasted the entire duration of pregnancy [3]. There is an increased incidence in patients with multiparity and elevated hormone levels of pregnancy [3]. Complications of the disorder include electrolyte imbalances and, rarely, fetal demise [3]. Management includes fluid repletion and antiemetic drugs such as first-generation antihistamines, but there is no specific treatment or preventative measure [1, 2]. Because HG is a diagnosis of exclusion, organic causes of gastrointestinal, genitourinary, metabolic, neurologic, psychologic, and pharmacologic sources must be considered during work-up [4]. Herein, we describe a case of a pregnant woman with HG who presented with intractable pain mimicking appendicitis.

## 2. Case Report

**Figure 1:**
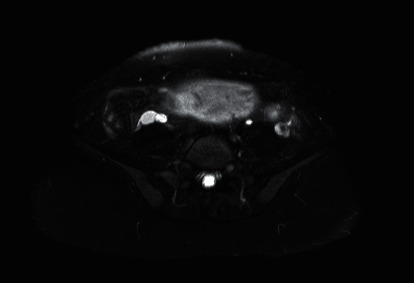
MRI of the abdomen depicting 12 mm uncomplicated appendicitis with surrounding inflammatory changes but no evidence of perforation.

A 33-year-old gravida 4 para 3 at 14 weeks' gestation with HG presented to the emergency department with several days of constant, sharp, 10/10 right back pain that radiated to her right flank, with associated nausea and vomiting, but no fevers, dysuria, abdominal pain, or spinal tenderness to palpation. Past medical history was significant for nephrolithiasis, abdominal vein thromboses, preeclampsia, bipolar I disorder, generalized anxiety disorder, and substance abuse. She was diagnosed with a probable nonobstructing 1 mm right kidney stone the day prior via an abdominal computed tomography (CT) scan at an outside hospital that did not visualize the appendix, and she reported similar symptoms with prior episodes of nephrolithiasis. Ultrasound (US) at presentation showed no hydronephrosis. She was admitted due to intractable pain and nausea. On admission, infectious work-up including blood culture and urine was negative. Urology was consulted and concurred that the severity of pain that the patient was experiencing was unlikely to be attributed to a kidney stone of that size. Labs at admission showed a normal white blood cell count of 7.7, but on day two of admission, labs were notable for significant leukocytosis to 20.0 (neutrophilia of 89.3) and mild hyponatremia. The leukocytosis decreased to 12.1 on day three and resolved to 9.7 on day four without intervention. Despite frequent doses of parenteral morphine and adjunctive pain medication, her pain was unremitting. Given pain worsened with vomiting, the suspicion arose that the pain was secondary to muscle spasm from frequent emesis, especially because her pain did not change with positioning, palpation, or movement. Obstetrics was consulted and optimized her antiemetic regimen for HG, while psychiatry managed her bipolar medication. Pain management was consulted to assist given the unrelenting nature of her pain and ever-increasing amounts of opiate requirement. At this time, the etiology appeared to be musculoskeletal given the exacerbation of pain with emesis, absence of leukocytosis, only moderately elevated inflammatory markers (C-reactive protein 55.8, erythrocyte sedimentation rate 31) which can be seen in pregnancy, negative blood cultures, and negative abdominal US to date. Further work-up was performed to rule out other causes. Magnetic resonance imaging (MRI) and magnetic resonance venography (MRV) without contrast of the abdomen and pelvis were performed and were significant for a 12 mm uncomplicated appendicitis with surrounding inflammatory changes but no evidence of perforation (Figure 1), as well as mild right pelviectasis at 4.9 mm. While leukocytosis remained resolved and blood cultures were negative, procalcitonin was elevated to 0.61 and the decision was made to start patient on IV piperacillin-tazobactam as surgical intervention was not recommended at this point in her pregnancy being borderline second trimester and the uncomplicated nature of her appendicitis. Over the following days, she began to exhibit symptoms of improvement. She completed six days of IV antibiotics and was discharged home with pain controlled and able to tolerate oral intake.

## 3. Discussion

To our knowledge, this is the first reported case of HG presenting as appendicitis. What is notable about our patient is that her intractable pain unresponsive to significant analgesia appeared to resolve with treatment for appendicitis, despite an uncharacteristic presentation with mildly consistent radiographic findings for such. Thereby, the detection and treatment of concurrent organic causes is important as it may provide significant symptomatic relief in cases of concurrent HG.

Imaging for appendicitis is typically performed with CT of the abdomen as this imaging modality has the lowest negative appendectomy rate (NAR) and is more diagnostically accurate [5]. However, given that our patient was pregnant, US was performed instead of repeating the CT abdomen as is the standard of care, given the risks of radiation to the fetus with CT. Since the US was not clear, a CT abdomen would have been considered next in work-up had she not been pregnant, as US is not a good modality for ruling out appendicitis [6]; historically, adding on a CT has prevented timely treatment [7]. Furthermore, it is well-supported that appendectomy is the preferred treatment of acute appendicitis in pregnancy [8–11] given concerns of rupture upon forgoing surgery [12]. A systematic meta-analysis by Lee, et al. showed no concern for fetal loss in laparoscopic appendectomy and no significant difference in surgical outcomes between laparoscopic and open surgery aside from decreased risk of wound infection and length of stay in the former [11]. However, the growing literature has documented the successful treatment of gestational appendicitis with antibiotics [13, 14]. While appendectomy remains first-line for appendicitis in pregnancy, this can serve as a useful tool for patients in rural areas who do not have access to immediate surgical treatment and need time to seek appropriate management [15]. Our paper does not aim to focus on appendicitis management but, rather, its varying presentations.

Although our patient had a significant psychiatric history, the consulted psychiatrist offered the patient risperidone, which she readily accepted as she was desperate for any adjunct therapies that could provide benefit. Therefore, there were no concerns of opiate-seeking in this case. Organic causes were strongly sought after, but there was no good evidence in her imaging studies for nephrolithiasis or appendicitis. As discussed in the case, the slightly elevated procalcitonin was enough to empirically treat for appendicitis since she was not responding to a maximized pain regimen, and her symptoms improved accordingly, which makes this case interesting. While pain in a patient with HG has a large differential including appendicitis [16], the atypical clinical presentation, mildly consistent radiographic findings, and mildly elevated inflammatory markers which can be normal in a pregnant person delayed early treatment. While it is difficult to rule in a diagnosis when it presents uncharacteristically, this case reiterates the significance of a thorough work-up and differential, as well as a strong clinical, suspicion. Also, in general, it can be difficult to differentiate a patient with appendicitis-like symptoms from one with true appendicitis, with CT/US providing no known increase in the accuracy of diagnosis [17]. Crucially, it is important to remember that HG is a diagnosis of exclusion, and efforts must be made to rule out other diagnoses, especially in cases of intractable pain that could be resolved without reliance on pain control.
